# Festival Dinner

**Published:** 1919-12-20

**Authors:** 


### GREAT NORTHERN CENTRAL HOSPITAL.

### FESTIVAL DINNER.

### Prince of Wales's Sympathy for Hospitals.

A SUCCESSFUL START TO THE FUND.

The festival dinner, given with the object of inaugurating a fund of £200,000 for the extension of the Great Northern Hospital, took place at the Connaught Rooms on Friday night, December 12. The Prince of Wales, who became President of the hospital in May of this year, presided over an extremely representative and distinguished assembly. Among those who were present were the Marquis of Northampton (Chairman of the hospital), the Duke of Argyll, the Duke and Duchess of Somerset, Marquis Camden (one of the Vice-Presidents of the hospital), General Lord Rawlinson, Admiral Sir Doveton and Lady Sturdee, Mr. H. J. Tennant (Deputy-Chairman of the hospital) and Mrs. Tennant, the Bishop of Willesden, Sir James and Lady Crichton-Browne, the Marquis of Dufferin and Ava, Lord Claud Hamilton, Sir Aston and Lady Webb, Mr. G. Lawson Johnston (the hospital's hon. treasurer), Lady Randolph Churchill, Lady Garvagh, General Sir Arthur Sloggett, and Mr. Horatio Bottomley. There were nearly five hundred guests.

Particularly evident throughout the dinner was the very genuine desire to help forward the invaluable and magnificent work being done by the Great Northern Hospital, and the very great affection in which the Prince of Wales is held. Perhaps it is well to set out in brief summary the hospital's astonishing record during the sixty-three years of its existence. Since its foundation in 1856 the Great Northern has treated 70,000 in-patients and over 1,250,000 out-patients. Its work has rapidly increased during recent years, as may be gauged from the fact that in the year 1918 alone there were 2,834 in-patients, 24,320 out-patients (new cases), while out-patient attendances numbered 123,500, and operations performed 1,403. The hospital contains ten special departments for the treatment of particular diseases, and the most advanced medical and surgical science is placed at the disposal of those poor people of North London who, in the main, comprise those who need its benefits.

The increased cost of commodities affects hospitals precisely in the same way as it affects individuals. At least £50,000 is now needed annually for maintenance to enable the hospital to carry on its everyday work. The facilities available at the hospital are quite inadequate for its needs, and there is frequently a list of 250 patients awaiting admission to the hospital. As the Prince pointed out, this may frequently result in the loss of lives which might have been saved were the accommodation of the hospital sufficient to allow of prompt admission, and, at the least, the inevitable tendency is very seriously to aggravate the maladies from which these would-be in-patients are suffering.

The most urgent provisions necessary to relieve this lamentable state of affairs—additions which it is hoped to make with the money raised for the Progress Fund—are: A block of four wards containing 100 beds; new departments for out-patients and accidents; additional wards for contributing patients; larger observation wards; extension of the *x*-ray department; additional operating theatres; and a nurses' home. With regard to the last item, the Great Northern nurses now live under extremely inconvenient conditions in "seven semi-detached villas," as Lord Northampton called them. "Indeed," said the Marquis, "if anyone is entitled to strike at the present time they are the nurses of the Great Northern Hospital"—an opinion which was greeted with hearty agreement.

It was quite evident from the two speeches made by the Prince that he realises very deeply the imperative need for placing the hospital in a better position to meet the demands upon it. There is something transparently genuine about the Prince. His enthusiasms are fresh and he feels deeply and does not conceal his feelings. It was very evident how impressed he was at the thought of those 250 urgent cases—sick men, women, and children needing immediate care and skilful nursing, and yet forced to wait weeks and sometimes months before proper attention became available to them. His mind seemed continually to revert to this picture of preventable suffering—he lost no opportunity of forcing it home to his hearers. That (he said in effect) is why we are here; not to make speeches, not to dine together, but to relieve the sufferings of the less fortunate, to bind up the wounds of those who have been struck down by a cruel fate. He played upon the compassion of his hearers; he came right down to essentials. When he proposed the toast of the evening, his speech was devoted to the work and the needs of the hospital; when he responded to the toast of his health he neglected entirely to refer to himself—he took the fresh opportunity of driving home his appeal for funds to relieve the suffering of those who clamour at the doors of the institution. He did not speak from carefully prepared notes; he spoke very genuinely from his heart. His words were no stilted words; he said simply and effectively what he felt, and his appeal, for this reason, was all the more effective.

The Prince has something more than the influence which is his by virtue of his position. The world, as one of the speakers said, loves him, not because he is a prince, but because he is himself. Of course, references were made to his wonderful tour in America, the amazing popularity which he had achieved, and the splendid results his visit must have had from an international point of view. Lord Camden quoted those charming words of the King, spoken to the Prince at the Buckingham Palace banquet held to celebrate his return: "Both your mother and I," the King had said, "are proud of you." Lord Camden added: "All of us—the whole Empire—can echo those words." Mr. Bottomley made the same point. "The Prince's visit," he said, "has done more to solve knotty international problems than all the politicians could have accomplished in twelve months in the House of Commons." Mr. Bottomley, incidentally, had one regret about the Prince's visit to the United States. "If only he could have waited there a few more days, I am quite sure," Mr. Bottomley said, "he could have talked to those recalcitrant Senators, persuaded them to sign the Treaty, and could have brought it back in his pocket to offer it here to the highest bidder for the benefit of the Great Northern Hospital!"

**Lord Northampton** had a typical story to tell of the Prince. When he was in France the Staff had great

**Great Northern Central Hospital—**(*continued*).

difficulty in preventing him from going over the top with his men in the Grenadier Guards. "Why shouldn't I go with them?" the Prince had demanded, indignantly. "I've got plenty of brothers if I get knocked out." Lord Camden gave a picture of the Prince in the hunting-field. "When the hounds are away," he said, "there's not a fence in the world that would stop him. And," he added, "there's nothing else that can stop him, either!"

After the Royal Toasts—which, incidentally, the Prince proposed at the time of the sorbet and not at the conclusion of the dinner, thereby establishing an interesting precedent—the toast of the "Imperial Forces" was proposed by Sir James Crichton-Browne, who made fitting references to the services of Sir Doveton Sturdee, the victor of the Falkland Isles battle, and of Lord Rawlinson, in whose Fourth Army the Prince of Wales served in France. Sir James also referred to the services of the Voluntary Hospitals during the war, and said it would be a great calamity if they were nationalised. "Some people seem to think," he said, "that the word 'nationalisation' holds more virtues than that blessed word 'Mesopotamia.'"

Mr. Bottomley supported the toast.

**Admiral Sturdee** and **Lord Rawlinson** replied, and the latter declared his willingness to do a little fighting and to make a sortie on behalf of the Great Northern.

In proposing "Success to the Great Northern Hospital Fund" **the Prince** recalled that "my family," had taken an active interest in the hospital for the last sixty or seventy years. He referred to the system of private wards which, although it at first provoked opposition, had since proved a very great boon to the community in providing aid to the middle classes, who could only afford to pay part of the cost of medical treatment. He also spoke with approval of the special departments for women and children which were going to be extended, and which were recognised as a centre for the North of London. He then went on to speak of the work of the hospital for ex-soldiers, and said there were sixty beds available for discharged men, and 100 men were being attended as out-patients. "I need not remind you," said the Prince, "of the great importance of what has now become our duty of looking after the men of all our three Services who fought and won in the war." Though the hospital has as many as 310 beds, it serves an area of 80 square miles, with a population of about a million, including several very poor districts.

People do not realise, continued the Prince, the difficulties that London hospitals have to contend with. More help is needed from the City and the West End and from the area in which the hospital is situated. The public must not allow the activities of the hospital to be impaired in any way. The Great Northern Hospital, on account of its situation, does not come before the public eye as much as some of the other hospitals, and for that reason he appealed to them not to forget this great institution. (Cheers.)

**Lord Northampton,** replying, said the need of extension is very great, for they cannot allow the discharged sailors and soldiers, and much less the poor, to go unattended and uncared for. Referring to the growth of the hospital, he recalled that in 1857 there was one nurse only, and the total salaries for nurses at that time was exactly 12s. weekly! In 1919 they had 125 nurses, costing over £40,000 annually. In 1910 the out-patients numbered 77,000; in 1919 they were estimated to have been over 150,000. In 1910 the hospital's expenditure was £19,000 annually; now it is £50,000. He mentioned that the Prince has promised shortly to pay a visit to the hospital. Mr. Tennant also replied.

Replying to the toast of his health, proposed by Lord Northampton, the Prince assured the gathering of his keen interest in hospitals, not only in London but throughout the Empire. He said that Lord Northampton had asked that one of the new wards of the hospital should be named after himself, and he would be very glad that this should be so. In conclusion the Prince gave the toast of "The Ladies," which was enthusiastically honoured.

It was announced that over £25,000 had been received towards the Hospital Progress Fund. The following contributions have been made :—The Prince of Wales, £50; Mr. C. P. Merriam, J.P., £1,500; King Edward's Hospital Fund for London, Mr. John Latta (in remembrance of members of the Baltic Shipping Exchange who made the supreme sacrifice), and the National Sunday League (per Mr. H. Mills), £1,000 each; Mr. Arthur Wheeler and Anonymous, £525 each; Lord Northampton (chairman of the hospital), Mrs. Edward Tufnell, Mrs. Rowcroft, 4 per cent. Victory Bonds (per Hon. Mrs. Burns, O.B.E.), Mr. H. G. Tetley, Mr. L. T. Bartholomew, Mr. Kennedy Jones, M.P., and Mr. James Fairbairn, £500 each; Mr. H. J. Tennant and Miss Emily Garrett, £300 each; Mr. E. G. Harrop and Messrs. Keith and Blackman, Ltd., £250 each; and Anonymous, £200.

